# Glucose Metabolism Disorders: Challenges and Opportunities for Diagnosis and Treatment

**DOI:** 10.3390/metabo12080712

**Published:** 2022-07-29

**Authors:** Jelena Vekic, José Silva-Nunes, Manfredi Rizzo

**Affiliations:** 1 Department of Medical Biochemistry, Faculty of Pharmacy, University of Belgrade, 11000 Belgrade, Serbia; jelena.vekic@pharmacy.bg.ac.rs; 2 Department of Endocrinology, Diabetes and Metabolism, Centro Hospitalar Universitário de Lisboa Central, 1069-166 Lisbon, Portugal; silvanunes2004@yahoo.com; 3 Department of Health Promotion, Mother and Child Care, Internal Medicine and Medical Specialties, University of Palermo, 90100 Palermo, Italy

Alterations of glucose metabolism are recognized as one of the most important risk factors for the development and complications of cardiometabolic diseases. The relationship between glucose metabolism disorders and cardiometabolic diseases is complex and mediated by multiple dysregulated pathways. Insulin resistance, the main driving force of disturbed glucose homeostasis, is strongly associated with obesity and metabolic syndrome [1]. In addition to metabolic disorders, which include impaired glucose tolerance, abdominal obesity, decreased high-density lipoprotein cholesterol (HDL-C) level and elevated triglycerides (TG), metabolic syndrome is characterized by a procoagulant, proinflammatory and prooxidant state [2], which further increases the likelihood of developing ischemic cardiovascular (CVD) and cerebrovascular diseases. However, biomarkers of these processes are seldom evaluated in clinical practice and therefore require further validation.

In general, an abnormal peripheral tissue insulin sensitivity and pancreatic β-cell dysfunction are presented even before glucose level exceeds the optimal thresholds, which is increasingly acknowledged as prediabetes [3]. Currently, there is a growing support for the approach that individuals with prediabetes should be closely monitored in order to recognize early subtle changes which might assist to delay or prevent development of diabetes. In addition to individuals in prediabetes state, there are also certain vulnerable groups with impaired glucose metabolism who require further considerations. Women with gestational diabetes mellitus are prone to develop serious pregnancy complications, including preeclampsia, preterm birth, macrosomia and neonatal hypoglycemia [4]. More recently it was documented that moderate hyperglycemia per se is associated with increased risk of pregnancy complications [5]. Moreover, a mounting body of evidence point towards increased risk for cardiometabolic disorders of both mother and a child in the future [6]. However, although available data supports the screening of cardiometabolic risk factors during pregnancy and postpartum [7,8], further improvements of screening strategies are required [9]. Apart from in utero predisposition, glucose metabolism disorders in childhood and adolescence, particularly type 1 diabetes mellitus, possesses a long-lasting impact on CVD risk [10,11]. In recent years, the increasing prevalence of type 2 diabetes mellitus in pediatric population was observed, as well as its strong association with childhood obesity, sedentary lifestyle and the western pattern diet [12]. These data suggests that timely recognition and optimal management of glucose metabolism disorders and accompanying risk factors could reduce both short-term and long-term consequences of gestational and childhood-onset diabetes. Taking into account the delicacy of this issue, additional longitudinal studies with comprehensive evaluation of biomarkers associated with glucose metabolism disorders in order to reveal early signs of subclinical atherosclerosis, are highly welcomed.

Despite well-known limitations of conventional biochemical parameters for the diagnosis and treatment monitoring, clinical validation of recently proposed candidates, such as advanced glycation end-products (AGEs), fructosamine and glycated albumin, is still pending [13]. Furthermore, the integration of innovative methods in medical laboratories, such as genomic, transcriptomic, epigenomic, proteomic and metabolomic techniques, enabled a more in-depth evaluation of disease-specific molecular pathways [14]. In that sense, metabolomics studies are of particular importance for identification of novel biomarkers related to glucose metabolism disorders [13]. These emerging biomarkers could also represent potential therapeutic targets in disease management, paving the way toward more personalized strategies. Nevertheless, the implementation of metabolomics biomarkers in clinical practice is faced with several limitations, the most important being restricted availability to the clinicians, unmet need for validation and harmonization of the assays and uncertain cost-effectiveness, which require further evaluation.

One of the most prominent mechanisms involved in complex etiology of microvascular and macrovascular complications of diabetes is dyslipidemia. It is now firmly established that worsening of metabolic control triggers disorders of lipid metabolism at multiple levels [15]. Diabetic dyslipidemia is characterized by qualitative changes of plasma lipoproteins, the most prominent being increase in small, dense low-density lipoprotein (LDL) particles [16,17]. In contrast with LDL, which size is inversely related to the atherogenic potential [18], there is still no reliable answer to the question on the relationship between physico-chemical and atheroprotective properties of HDL particles in the conditions associated with high CVD risk [19]. Analysis of HDL proteome and metabolome constitutes a promising filed which could provide valuable information on the relationship between the structure and function of this lipoprotein in high-risk patients [20].

In order to comprehensively present all aspects of disturbed glucose homeostasis, it is important to consider potential effects of anti-diabetic therapeutics on hyperglycemia biomarkers and associated risk factors. Data from large epidemiological studies and clinical trials have provided significant evidence that lowering fasting and post-prandial glycemia, as well as ameliorating other indices of glucose metabolism alterations, such as glycated hemoglobin (HbA_1c_), is an effective approach to reduce cardiometabolic risk [21]. In light of these findings, reduction of glycemic excursions is increasingly important aspect of diabetes management. However, different metrics were used to assess glycemic variability across the studies [21] and more data is needed to define the most useful indices in clinical practice. In addition, it is equally important to assess the efficacy of glycemic fluctuations control among different anti-diabetic medications and combined therapy. To date, a variety of pharmaceutical and nutraceutical approaches are available for management of diabetes and its co-morbidities. Notably, innovative anti-diabetic agents have a broad spectrum of cardioprotective mechanisms, which goes far beyond their hypoglycemic effects [22,23]. It is therefore critically important to further address which biomarkers, alone or as a multimarker panel, are the most suitable for monitoring these pleiotropic effects of novel anti-diabetic medications.

By overcoming the COVID-19 outbreak, diabetes mellitus remains the largest epidemics of the world. In the following period, it should not be neglected that diabetes is a major contributor to COVID-19 severity and mortality, as well as that the maintenance of optimal glycemic control is the key to reduce the risk from infection [24,25]. A growing trend of post-COVID patients urges the need to increase awareness about the risk of new-onset hyperglycemia or diabetes, in attempting to prevent its adverse consequences [26]. As stated above, a number of potential biomarkers of glucose metabolism disorders have been identified, but they have not yet been sufficiently explored in the post-COVID era.

This Special Issue of *Metabolites* is aimed to critically review available evidence from basic science and clinical studies on the above listed challenges that need to be met, in order to contribute to a better understanding of glucose metabolism alterations and their effective management and prevention.
